# Gene expression differences in *Longissimus* muscle of Nelore steers genetically divergent for residual feed intake

**DOI:** 10.1038/srep39493

**Published:** 2016-12-22

**Authors:** Polyana C. Tizioto, Luiz L. Coutinho, Priscila S. N. Oliveira, Aline S. M. Cesar, Wellison J. S. Diniz, Andressa O. Lima, Marina I. Rocha, Jared E. Decker, Robert D. Schnabel, Gerson B. Mourão, Rymer R. Tullio, Adhemar Zerlotini, Jeremy F. Taylor, Luciana C. A. Regitano

**Affiliations:** 1 Embrapa Pecuária Sudeste, São Carlos, SP, Brazil; 2 Division of Animal Sciences, University of Missouri Columbia, Columbia, MO, USA; 3 Department of Animal Science, University of São Paulo/ESALQ, Piracicaba, São Paulo, Brazil; 4 Department of Genetics and Evolution, Federal University of São Carlos, São Carlos, SP, Brazil; 5 Informatics Institute, University of Missouri, Columbia, Missouri, 65211, USA; 6 Embrapa Informática Agropecuária, Campinas, SP, Brazil

## Abstract

Residual feed intake (RFI), a measure of feed efficiency (FE), is defined as the difference between the observed and the predictable feed intake considering size and growth of the animal. It is extremely important to beef production systems due to its impact on the allocation of land areas to alternative agricultural production, animal methane emissions, food demand and cost of production. Global differential gene expression analysis between high and low RFI groups (HRFI and LRFI: less and more efficient, respectively) revealed 73 differentially expressed (DE) annotated genes in *Longissimus thoracis* (LT) muscle of Nelore steers. These genes are involved in the overrepresented pathways Metabolism of Xenobiotics by Cytochrome P450 and Butanoate and Tryptophan Metabolism. Among the DE transcripts were several proteins related to mitochondrial function and the metabolism of lipids. Our findings indicate that observed gene expression differences are primarily related to metabolic processes underlying oxidative stress. Genes involved in the metabolism of xenobiotics and antioxidant mechanisms were primarily down-regulated, while genes responsible for lipid oxidation and ketogenesis were up-regulated in HRFI group. By using LT muscle, this study reinforces our previous findings using liver tissue and reveals new genes and likely tissue-specific regulators playing key-roles in these processes.

Feed efficiency is an important trait to cattle production systems worldwide because of the relationship between an individual’s efficiency and income over feed costs. Different measures of feed efficiency have been proposed^1^ and most of these traits have been reported to have at least moderate heritability in beef and dairy cattle^2^,^3^,^4^,^5^,^6^, including estimates in our population from the Nelore breed^7^.

The selection of animals with higher potential genetic merits for feed efficiency is an important strategy to reduce demands for feedstuff and production costs, thus increasing industry competitiveness. Furthermore, the selection of efficient animals may reduce the land area required for production, as well as methane emissions and nitrogen excretions resulting from metabolic processes^1^,^8^. Consortia have performed studies worldwide to estimate the genetic (co)variation underlying feed efficiency related-traits to develop alternative opportunities for the genetic evaluation of feed efficiency. As a result, potential biomarkers from gene expression studies, conducted in beef cattle divergent for Residual Feed Intake (RFI), have now been proposed^9^,^10^. The relationship between growth rate and gross feed efficiency has been exploited in national breeding programs by selecting for increased growth rate. However, this approach can lead to an increase in maintenance energy costs in mature females^1^. To overcome this, several indices have been proposed to estimate feed efficiency independently of growth rate. Residual feed intake is used to identify individuals that deviate from the average feed intake of animals at the same average daily gain (ADG) and metabolic mid-weight (MMW; mid-test weight 0.75). Consequently, animals with higher RFI are less efficient at converting feed into body mass regardless of their growth rates.

Genome-wide association studies (GWAS) have widely been used as a strategy for investigating mutations underlying economically important traits in livestock. Several candidate loci and genes associated with feed efficiency traits in Nelore cattle have been identified by these GWAS^7^,^11^. Most of the loci identified in these studies appear to be located in regulatory elements and may affect phenotype by contributing to variation in the expression levels of nearby key-genes. Current technologies allow the measurement of the expression of each gene in each tissue of an individual using, for example, RNA sequencing (RNA-seq). Recently an RNA-seq study reported that changes in the hepatic gene expression between efficient and inefficient Nelore cattle primarily appear to be related to metabolic processes underlying oxidative stress^12^. This study identified several differentially expressed (DE) genes involved in mitochondrial functions. Supporting these results, studies have suggested that oxidative stress is increased in less efficient animals due to differences in mitochondrial function^13^. Feed efficiency is a complex trait involving many processes and organs. For example, skeletal muscle metabolism has a key role in resting energy expenditure^14^. Furthermore, by analyzing *Longissimus thoracis* (LT) tissue, evidence was provided that mitochondrial respiration rate was increased in the most efficient Angus animals^15^. Thus, numerous physiological mechanisms may be important to variation in feed efficiency and component traits, including cellular ATP synthesis, basal metabolic rate, regulation of growth and development, and the homeostatic control of body mass^16^.

Gene expression changes in the LT muscle of Nelore steers genetically divergent for RFI were detected by RNA-seq in 20 animals comprising two groups selected for their low or high estimated additive genetic merits for RFI (LRFI and HRFI, respectively). RNA-seq allows the examination of genome-wide gene expression differences between contrasting treatment groups and is repeatable, highly sensitive and strongly correlated with quantitative PCR experiments^17^. In this study, we hypothesized that gene expression differences in the LT muscle of animals with extreme differences in their best linear unbiased predictions of additive genetic merit for RFI could partially explain the differences in the feed efficiency phenotypes of these Nelore beef cattle.

## Results

### Sequencing throughput, read mapping, and assembly of transcripts

The feed efficiency phenotypes for this Nelore population had previously been used to perform a GWAS and the summary statistics for the population have previously been reported^7^. Information on backfat thickness (BFT) and intramuscular fat (IF) content for these animals has also previously been published^18^,^19^. It is known that RFI is related to fat deposition, including in this Nelore population^20^ and because of this a student’s t-test was applied to evaluate the mean differences in BFT and IF between the divergent RFI groups but no significant differences were observed (P ≥ 0.05). Summaries for estimates of additive genetic merit, phenotypes, sequencing throughput and mapping statistics for each sample used in this study are presented in Table 1. RNA-seq data were successfully generated for all 20 animals, with an average of 18,352,638 million reads per sample. On average, 90.82 ± 3.05% of reads were mapped to the *Bos taurus* UMD3.1 reference genome.

After mapping reads with TopHat v2.0.6^21^, Cufflinks v2.0.2^22^,^23^ was used to assemble the LT muscle transcriptome for each sample. The Cuffmerge utility was then run to create a unique file that contained a parsimonious set of non-redundant transcripts representing these data and Cuffcompare was used to compare the produced assembly to the reference annotation. We assembled 87,850 transcripts representing 26,525 genomic loci, of which, 80,830 were multi-exon transcripts. Of these loci, 17.5% were classified as novel. We found a total of 18,332 genes to be expressed in bovine muscle; however, 4,797 of these genes could not be tested because of their low expression.

### Differentially expressed genes and pathways related to feed efficiency

We identified 84 DE genes (q-value ≤ 0.05) between the HRFI (less efficient) and LRFI (more efficient) groups, with 73 of these being annotated genes (Table 2). Cytochrome P450 family 1 subfamily A member 1 (*CYP1A1*) was the only DE gene located within a quantitative trait locus (QTL) region identified by our previous GWAS for feed efficiency-related traits (explaining 0.29% of the additive genetic variance in RFI)^7^. BLAST searches against the NCBI non-redundant nucleotide sequence database were performed for the unannotated transcripts identified as DE. The majority of the unannotated DE transcripts shared significant similarity with non-coding RNAs (ncRNA) such as ribosomal RNAs (Supplementary Table S1). Non-coding RNAs function through several mechanisms that regulate posttranscriptional processes. Besides ncRNAs, we found a transcript that shared a significant homology with members of the mitochondrial D-loop gene family.

Among the DE genes, the highest fold change estimate was for the beta 3-glucosyltransferase (*B3GALTL*) gene, involved in the fucose metabolic process, which was up-regulated in the less efficient, HRFI animals. On the other hand, the lowest fold change estimates were observed for genes encoding ribosomal proteins. Because the 73 annotated genes included several that encode hypothetical proteins, only 59 genes could be used in the functional annotation analysis. The functional analysis using both the up- and down-regulated genes was performed using the Database for Annotation, Visualization, and Integrated Discovery (DAVID) v6.7^24^ and revealed that these DE genes are related to the metabolism of Xenobiotics by Cytochrome P450 family members (p ≤ 0.03) and Butanoate and Tryptophan Metabolism (p ≤ 0.1). In the metabolism of Xenobiotics by Cytochrome P450 pathway we found members of the cytochrome P450 family (cytochrome P450 family 1 subfamily B member 1 (*CYP1B1*) and *CYP1A1* and glutathione S-transferase pi 1 (*GSTP1*) genes to be DE between the feed efficiency groups. With the exception of *CYP1B1*, which was up-regulated in the less efficient, HRFI group, the other genes involved in this pathway were down-regulated in this group. The *CYP1B1* and *CYP1A1* genes are also involved in the Tryptophan Metabolism pathway. On the other hand, 3-hydroxybutyrate dehydrogenase, type 1 (*BDH1*) and acyl-CoA synthetase medium-chain family member 1 (*ACSM1*) which are involved in butanoate metabolism, were up-regulated in the less efficient, HRFI group.

The summarized Biological Processes from the Gene Ontology provided by DAVID revealed many important processes underlying feed efficiency variation in Nelore cattle, such as response to organic substance, release of cytochrome c from mitochondria and macromolecule catabolic process (Table 3). The enriched functional cluster included metal ion binding, negative regulation of apoptosis, response to organic substance, transcription regulator activity and intracellular organelle lumen and glycoprotein (Supplementary Table S2). We found *BDH1*, coenzyme Q10B (*COQ10B*), NADH:ubiquinone oxidoreductase core subunit S7 (*NDUFS7*), *ACSM1*, cellular myelocytomatosis oncogene (*MYC*) and *CYP1A1*, related to mitochondrial functions to be among the DE genes. Furthermore, genes with a recognized role in feed efficiency such as Arrestin Domain Containing 3 (*ARRDC3*)^25^ and *GSTP1*^9^ were detected to be DE, supporting our results. These findings are supported by our previous study investigating global liver gene expression changes in Nelore steers from the same population, where 18 animals sampled as divergent RFI phenotypes^12^ were common to this study.

**Figure 1:**
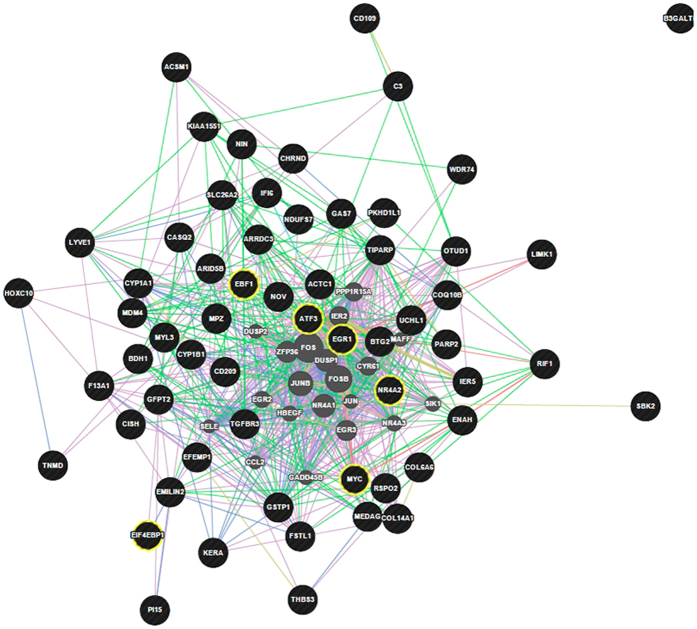
Network integration of differentially expressed genes between the efficient (LRFI) and inefficient (HRFI) groups. Genes presented as black circles were differentially expressed (DE) between the efficient (LRFI) and inefficient (HRFI) groups. Genes presented in grey interact with the DE genes. Circles highlighted in yellow are transcription factors. Arrows in pink, green, blue and red represent co-expression relationships, genetic interactions, co-localizations and physical interactions, respectively.

**Figure 2:**
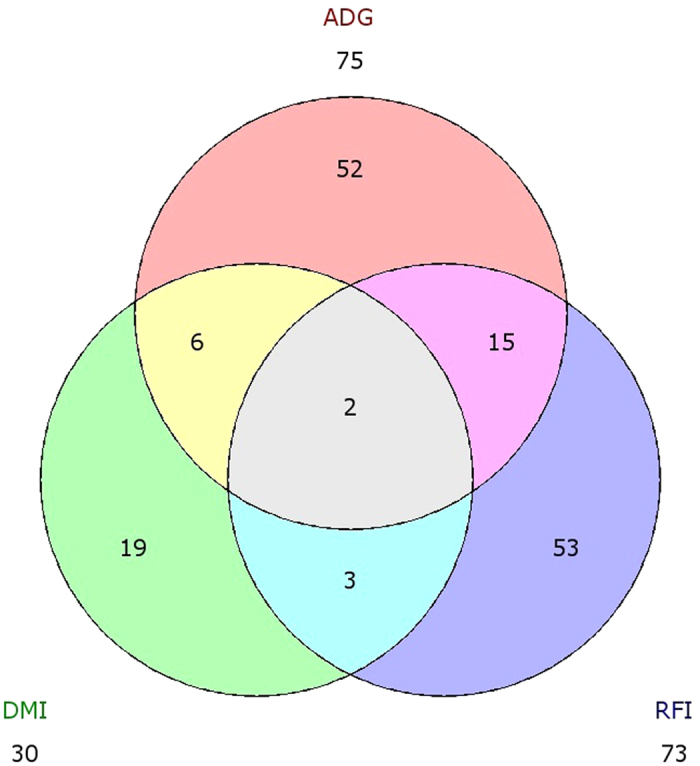
Network integration of the *EGR1* gene and other differentially expressed genes. Genes presented as black circles were differentially expressed (DE) between the efficient (LRFI) and inefficient (HRFI) groups. Genes presented in grey interact with the DE genes. Arrows presented in pink, green, blue and red represent co-expression relationships, genetic interactions, co-localizations and physical interactions, respectively.

We predicted using the GeneMANIA prediction server^26^ that several transcription factors such as *MYC*, Activating Transcription Factor 3 (ATF3), Nuclear Receptor Subfamily 4, Group A, Member 2 (*NR4A2*) and Eukaryotic Translation Initiation Factor 4E-Binding Protein 1 (*EIF4EBP1*) were responsible for expression changes detected in this study (Fig. 1). The up-regulation of transcription factors known to be induced under oxidative stress was found in the less efficient, HRFI group. For example, the network integration analysis showed that *EGR1*, known to induce “redox sensitive” genes^27^, interacts with several of the DE genes (Fig. 2). The network integration of DE genes between the LRFI and HRFI groups (Fig. 1) shows how the DE genes may interact. The constructed network revealed that 78.56% of the component genes are known to be co-expressed in humans. Furthermore, 9.02% are co-localized genes (i.e., genes expressed in the same tissue and/or proteins that are found in the same cellular location), 7.86% are known to physically interact, 2.88% are known to have genetic interactions, 1.33% share protein domains, and finally, 0.35% are related to common pathways.

**Figure 3:**
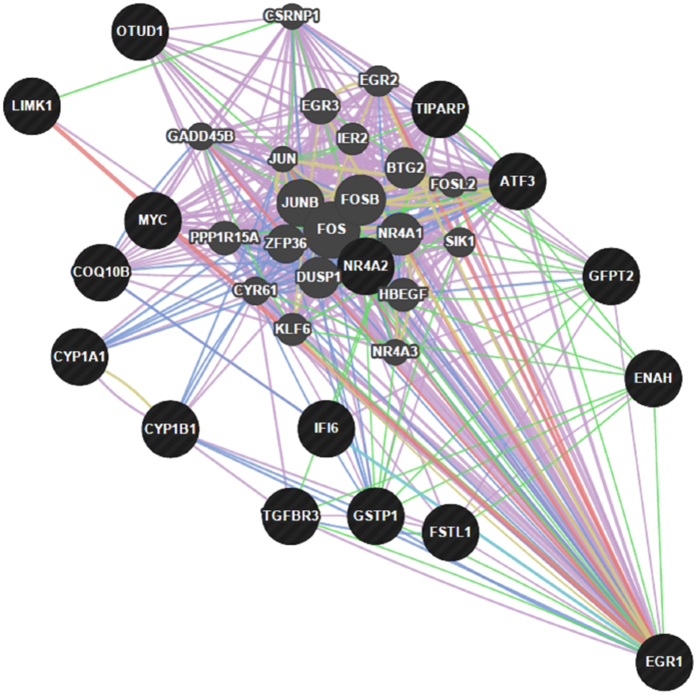
Venn diagram showing overlaps between differentially expressed genes found for residual feed intake (RFI), average daily gain (ADG) and dry matter intake (DMI).

Dry matter intake (DMI) and average daily gain (ADG) were used to decompose RFI for additional gene expression analyses. This was accomplished by regrouping the same animals used for the RFI analysis based on their phenotypes for these traits. Of the 75 DE annotated genes for ADG (Supplementary Table S3), 17 were also identified for RFI and of the 30 DE genes for DMI (Supplementary Table S4), 5 genes were also DE for RFI (Fig. 3). On the other hand, DMI and ADG shared 6 DE genes (Fig. 3). Actin, alpha, cardiac muscle 1 (*ACTC1*) and elongation factor 1-alpha 1 pseudogene (*LOC782776*) were found to be DE in all analyses. The common genes found to be DE for both RFI and ADG were activating transcription factor 3 (*ATF3*), mesenteric estrogen-dependent adipogenesis (*MEDAG*), collagen type VI alpha 6 (*COL6A6*), *CYP1B1*, collagen type XIV alpha 1 (*COL14A1*), EGF containing fibulin-like extracellular matrix protein 1 (*EFEMP1*), follistatin-like 1 (*FSTL1*), immediate early response 5 (*IER5*), uncharacterized *LOC100335754*, lymphatic vessel endothelial hyaluronan receptor 1 (*LYVE1*), *MYC*, nephroblastoma overexpressed (*NOV*), OTU deubiquitinase 1 (*OTUD1*), poly (ADP-ribose) polymerase 2 (*PARP2*) and peptidase inhibitor 15 (*PI15*). DE genesMHC class II antigen (*BLA-DQB*), elongation factor 1-alpha, somatic form-like (*LOC100300305*) and uncharacterized *LOC100848726* were DE for both DMI and RFI. Among the DE genes for ADG, we observed adiponectin, *C1Q* and collagen domain containing (*ADIPOQ*) and thrombospondin 1 (*THBS1*), related to response to carbohydrate stimulus and regulation of lipid transport, in addition to fatty acid binding protein 4, adipocyte (*FABP4*) which is involved in the regulation of brown fat cell differentiation (Supplementary Table S5). These genes were up-regulated in the high-ADG group, moreover, *FABP4* was also found to be up-regulated in the high DMI group. Finally, Regulator of G-protein signaling 2 (*RGS2*), which had already been identified as regulating feed efficiency in this population^12^, was down-regulated in the high-DMI group. Likewise, Niemann-Pick disease, type C2 (*NPC2*) and FBJ osteosarcoma oncogene (*FOS*) genes, which are related to lipid transport and response to reactive oxygen species (ROS), respectively, were up-regulated in the high-DMI group.

## Discussion

The productivity of beef cattle is highly influenced by the rate of conversion of feed to muscle growth resulting in greater meat yields per unit of input. Therefore, any improvement in an animal’s efficiency that does not negatively affect other profitability-related traits, such as fertility or disease resistance, will result in increased herd profitability. These traits have been shown to be heritable in Nelore cattle and possess sufficient genetic variation to be valuable for selection^7^. The selection of efficient animals is an important approach for the improvement of herd profitability; however, the collection of individual animal feed intake data on large samples of animals is expensive and logistically impractical. Variation in total energy expenditures of animals of the same breed and under similar management circumstances may arise from differences in physiological processes such as ion pumping, mitochondrial proton leak, uncoupling protein activities, thyroid hormones, leptin, IGF-1 and lipid metabolism^28^. Gene expression is a key source of variation between individuals and may be used to identify functional candidate genes and pathways that control target traits. The aim of this study was to elucidate the gene expression changes in LT muscle of Nelore cattle differing in RFI to identify putative pathways controlling variation in this trait. Also, because we had previously performed a gene expression study in the liver tissues^12^ of these animals, we highlighted the similarities and differences found between the different tissues.

Most of genes found to be DE in this study do not reside within previously identified QTL regions for RFI that were found in a GWAS study using animals from the same Nelore population^7^, with the exception of the *CYP1A1* gene; however, several common biological mechanisms were detected, such as those related to lipid and fatty acid (FA) metabolism. In fact, QTL regions do not necessary have to harbor DE genes, since QTL can be created by mutations in genes that cause post-translational disruptions affecting the functionality of proteins. Furthermore, the majority of QTLs identified in the GWAS performed for this Nelore population lay within gene deserts^7^ and therefore may affect feed efficiency via effects on regulatory elements, such as distal enhancers and transcription factor binding sites, which are poorly characterized in cattle.

The highest fold change estimate was found for *B3GALTL*, which is involved in fucose metabolic process and functions as an O-glucosyltransferase, contributing to the elongation of O-fucosylglycan. Genes related to fucose metabolism have previously been identified as playing a role in feed conversion efficiency^29^. Genes induced by fucose have been suggested as being important for cross-talk between the intestinal microbiome and host tissues in mice^30^ and rabbits^31^. For example, Fucose Sensing Kinase (*FusK*) senses fucose and controls the expression of metabolic genes^31^. On the other hand, among the DE genes, the lowest fold changes were systematically observed for genes related to translational regulation and ribosome biosynthesis. Ribosomal protein genes may provide a starting point for better understanding gene regulation and for constructing gene regulatory networks related to different biological functions^32^. The cellular energy status is an extremely dynamic variable that contributes to metabolic fluctuations that can affect the rates of ribosome biogenesis^33^. Furthermore, ribosome biogenesis may have an essential role in the regulation of skeletal muscle mass^34^.

Genes that have already been reported as functioning in the regulation of feed efficiency-related traits were identified. For example, *ARRDC3*, up-regulated in the less efficient group, plays a role in the regulation of metabolism and obesity in humans and mice^25^. The fed-to-fasting transition period plays an important role in the regulation of metabolic activity. A study with mice has shown that *ARRDC3*-deficiency protects against obesity by increasing energy expenditure likely acting by increasing β-adrenergic signaling to stimulate thermogenesis in brown and white adipose tissues^25^. Skeletal muscle is an important organ for thermogenesis and resting energy expenditure. Also, the investigation of DE genes implicated in either fed or fasting gene-expression in human adipose tissue has been performed and *ARRDC3* was found to be among the 10 most responsive genes to food intake, with higher transcript levels during periods of fasting^25^.

The functional analysis using both the up- and down-regulated genes performed by DAVID revealed that metabolism of Xenobiotics by cytochrome P450, butanoate and tryptophan metabolism pathways influence RFI in Nelore cattle. Cytochrome P450 enzymes are responsible for hydrolysis, oxidation or reduction mechanisms of xenobiotic metabolism^35^. These enzymes catalyze many reactions involved in drug metabolism as well as the synthesis of cholesterol, steroids and other lipids^36^. If these mechanisms are insufficient to clear a compound from circulation, or if the compound generates a reactive metabolite, glutathione S-transferases act as endogenous antioxidants. We found two DE members of the cytochrome P450 family involved in the metabolism of Xenobiotics by Cytochrome P450 pathway as well as in the Tryptophan Metabolism pathway. Besides the genes that directly encode for cytochrome P450 enzymes, we found *GSTP1* functioning in the metabolism of Xenobiotics by Cytochrome P450 pathway to be DE between the feed efficiency groups. These genes were primarily down-regulated in the less efficient, HRFI animals, and may indicate that the malfunction of these mechanisms may result in an excess of ROS production or in a decrease in ROS reduction capacity, which in turn can cause lipid peroxidation and oxidative stress^37^. Agreeing with the results observed in this study, the down-regulation of *CYP1A1* has been reported in inefficient Yorkshire pigs^38^. This gene is located in a previous defined RFI QTL region identified in this Nelore population^7^.

Mitochondrial dysfunction and oxidative stress have been implicated as major processes underlying variation in feed efficiency. How mitochondrial functions are associated with feed efficiency in livestock has recently been addressed^13^,^39^,^40^,^41^. Mitochondria play a major role as energy suppliers and ROS regulators^42^. Furthermore, other studies with cattle^43^,^44^, including our previous study investigating global liver gene expression changes in Nelore steers from this population^12^ demonstrate that less feed efficient animals exhibit gene expression differences that are related to the modulation of conditions of oxidative stress.

Mitochondrial activity may be compromised under oxidative stress. *NDUFS7* was found to be down-regulated in the less efficient group. The protein encoded by this gene is a subunit of one of the complexes that forms the mitochondrial respiratory chain, which functions in the transfer of electrons from NADH to the respiratory chain. Supporting our findings, differences in oxygen consumption and oxygen pulse were detected when comparing more and less efficient animals of this population. Oxygen consumption per heart beat was lower for more efficient steers, and consequently, oxygen volume and oxygen pulse were also significantly lower^45^. A study has shown that electron transport chain (ETC) complex activities in the mitochondria of muscle, liver, and duodenal cells in inefficient broilers may be impaired^46^. These findings are reinforced by previous studies that suggested an association between compromised respiratory complex activities and increased ROS production^47^,^48^. Moreover, positive correlations between glutathione and activities of mitochondrial respiratory complexes II, IV, and V, indicate that antioxidant protection is important to the optimal activity of the ETC^46^. A consistent finding in all broiler studies was the evidence of increased protein oxidation in tissues obtained from broilers with low feed efficiency^48^. It is important to notice that although we observed several nuclear-encoded mitochondrial genes, no gene from the mitochondrial genome was detected to be differentially expressed.

We also found the Butanoate Metabolism pathway to be enriched for DE genes in our analysis. Butanoate is a metabolite of gut flora-mediated fermentation of dietary fiber and is closely involved with energy metabolism^49^. There is evidence that butanoate metabolism may be activated under conditions of cellular oxidative stress^49^. The genes *ACSM1* and *BDH1* that are involved in FA oxidation and ketogenesis are members of this pathway and were found to be up-regulated in the less feed efficient animals. Oxidative stress may reprogram lipid metabolism, attenuating lipid synthesis and increasing mitochondrial fatty FA oxidation^50^. In mice, high fat diet-induced obesity also correlates with mitochondrial dysfunction and increased oxidative stress in skeletal muscle and liver^51^. Moreover, high fat diets increase indices of lipid and protein oxidation in rodent hearts and also increase markers of apoptosis^52^. Oxidized lipids are known to trigger the transcriptional induction of *NR4A* gene family members^53^; in this study, *NR4A2* was found to be up-regulated in the less feed efficient animals.

The protein encoded by *BDH1* catalyzes the interconversion of two major ketone bodies produced during FA catabolism. Ketogenesis occurs primarily in the mitochondrial matrix of liver cells and is important because it provides energy for surrounding tissues, especially cardiac and skeletal muscle. Ketogenesis is the last step of lipid energy metabolism, a pathway that links dietary lipids and adipose triglycerides to the mitochondrial respiratory chain^54^. Oxidative stress may be an outcome of increased contact with oxidants, from reduced feed intake, de novo synthesis or increased turnover of antioxidants. The relationship between oxidative stress and metabolic disorders has been demonstrated in cattle by reducing antioxidant status during periods of ketosis and milk fever^55^. Cells must adapt their metabolism to produce all of the molecules and energy that is required under conditions of oxidative stress to survive. Less efficient individuals overexpressed genes related to the catalysis of lipids. In support of our results, it has already been shown that the more efficient Nelore steers of this population had significantly lower liver and internal fat proportions and lower extracted amounts of intramuscular fat compared to the less efficient steers^20^. Also supporting our findings, a study observed differentially co-expressed genes related to fatty acid metabolic processes in Nelore animals divergent for RFI, indicating the differential metabolism of lipids between these divergent feed efficiency groups^44^.

Moreover, mitochondria-generated ROS play an important role in the release of cytochrome c and other pro-apoptotic proteins, which can trigger caspase activation and apoptosis^56^. The Gene Ontology terms (GO) reported by DAVID indicated that DE genes, including *MYC* and *IFI6*, both up-regulated in the less efficient group, are involved in these functions. These genes have transcriptional activation functions and may regulate genes functioning in the mitochondrion and in mitochondrial respiratory chain complex activities and macromolecule catabolic processes. MYC family is known to be a proto-oncogene that activate genes involved in ribosomal and mitochondrial biogenesis, glucose and glutamine metabolism, lipid synthesis, and cell-cycle progression^57^.

Other genes with transcriptional activities were also found to be DE between the RFI groups. These genes may explain part of the expression changes that were observed in the comparison of the efficient and inefficient steers. Transcription factors, known to be induced under conditions of oxidative stress were found to be DE and up-regulated in the less feed efficient animals. Among these, we found *ATF3*, a member of the ATF/cAMP-responsive element-binding protein family of transcription factors that is known to be responsive to stress and ROS^58^. Additionally, expression of *ATF3* in the liver results in defects in glucose homeostasis by repressing gluconeogenesis^59^. Increased amounts of oxidizing agents stimulate signaling pathways. Besides the genes and pathways already discussed, we also identified early growth response 1 (*EGR1*), known to encode a zinc finger transcription factor that activates “redox sensitive” genes^27^. Our network integration analysis shows that *EGR1* interacts with several other DE genes related to oxidative stress, such as *GSTP1, NR4A2* and *CYP1A1*, for which functions have already been discussed. Our previous genome-wide gene expression study conducted on liver tissues indicated that gene expression changes between more and less efficient animals may define conditions of oxidative stress, indicating *EGR1* as a potential upstream regulator of these gene expression changes^12^.

It has also been demonstrated that the more efficient animals from this population possessed larger *longissimus* muscle areas, suggesting greater muscle deposition in the carcasses of efficient Nelore steers from the same population that was used in this study^20^. The expression of Myosin light-chain genes, related to muscle development, including *MYL3*, partially contributes to the difference in skeletal muscle deposition between duck breeds^60^. It is important to highlight that, via the regrouping of the same animals used for the RFI analysis based on their phenotypes for ADG and DMI, we performed additional DE gene analyses. Our functional analysis provided by DAVID indicated that high-ADG is related to increased response to carbohydrate, regulation of lipid transport and brown fat cell differentiation. Also of interest, genes related to lipid transport and response to ROS were up-regulated in the high-DMI group, as they were for the less efficient animals in the primary analysis. Accordingly, increased DMI was reported for the less efficient Nelore steers from this population^20^.

Feed efficiency is a complex trait involving many processes and organs. While skeletal muscle metabolism has a key role in resting energy expenditure, liver is extremely important in metabolizing carbohydrates, lipids, and proteins into biologically useful materials, processes that produce various compounds that generate reactive oxygen radicals and may cause oxidative stress. Skeletal muscles are densely populated with mitochondria: a main producer of ATP and FA oxidation. Mitochondrial dysfunction and FA oxidation are also implicated in oxidative stress. Among the DE transcripts were several genes related to mitochondrial function and the metabolism of lipids. Our studies indicate a role for DE genes related to mitochondrial function and lipid metabolism in liver^12^ and skeletal muscle related to feed efficiency phenotypes. By using LT muscle, the present study reinforces previous findings in liver^12^ that gene expression changes in Nelore cattle genetically divergent for RFI are related to metabolic pathways underlying oxidative stress. However, only 4 genes found to be DE in our previous analysis were also identified as DE here, indicating that new genes and likely tissue-specific regulatory patterns play key-roles in these processes. Several TF and genes with known functions in lipid metabolism and growth were also identified. These DE genes can be investigated to implement molecular strategies to improve feed efficiency. We also found new mechanisms, such as those related to fucose metabolism, as influencing feed efficiency that had not been identified in our previous work performed with liver samples.

In summary, we detected gene expression differences in the LT muscle of Nelore steers genetically divergent for residual feed intake. Our results support the hypothesis that gene expression differences between efficient and inefficient Nelore steers are primarily related to oxidative stress. Oxidative stress may arise when cellular mechanisms that reduce cellular ROS are compromised. In response, cellular transcription networks, such as metabolism of Xenobiotics by cytochrome P450, are altered. The results of this study, together with previous reports, provide a more comprehensive picture of gene expression in the muscle of animals genetically divergent for RFI. Further studies are necessary to dissect the mechanisms behind oxidative stress-induced signaling, which may help develop strategies to improve feed efficiency in beef cattle. Studies targeting the identification of functional mutations in elements regulating these DE genes may be important for the implementation of genomic selection for feed efficiency in Nelore cattle.

## Methods

### Animals and sampling

Experimental procedures were carried out in accordance with the relevant guidelines provided by the Institutional Animal Care and Use Committee Guidelines of the Embrapa Pecuária Sudeste – Protocol CEUA 01/2013. The Ethical Committee of the Embrapa Pecuária Sudeste (São Carlos, São Paulo, Brazil) approved all experimental protocols (approval code CEUA 01/2013). The steers used in the expression study (n = 20) were born and raised at Embrapa Pecuária Sudeste (São Carlos, São Paulo, Brazil) as described elsewhere^7^,^12^. They were allocated to feedlots at about 21 months of age. Within the feedlots, animals were maintained either in individual or collective pens and allowed ad libitum access to feed and water. Details were previously provided^7^.

The fed population of steers comprised half-sib families produced by the artificial insemination of commercial and purebred Nelore dams, derived from sires representing the main breeding lineages commercialized in Brazil. BLUP estimates of genetic merit for RFI were initially generated for 585 Nelore steers^12^. For the gene expression studies, we selected the 10 high- and 10 low-efficiency steers with divergent BLUP estimates of their additive genetic merits for residual feed intake RFI (kg/d). Where possible, animals that had common sires were sampled from each tail of the distribution of predicted genetic merits for feed efficiency. A student’s t-test was applied to evaluate if there were significant differences between the selected groups for BFT and IF.

### RNA sequencing

*Longissimus thoracis* (LT) muscle samples were collected from the ribeye steak at slaughter and immediately stored in liquid nitrogen. The samples were then kept at −80 °C until RNA extraction. Total RNA was extracted from 50–100 mg of muscle tissue using Trizol (Invitrogen^®^), following the manufacturer’s protocol. The total RNA concentration was measured by spectrophotometry, and quality was verified initially by the 260:280 ratio, followed by assessment of integrity by agarose gel electrophoresis. Only intact samples, with a RNA 260:280 ratio greater than 1.8, were sent for sequencing. Before sequencing, eight samples were randomly chosen to double-check RNA quality on the Agilent 2100 Bioanalyzer System. The RNA Integrity Number for all samples was higher than 7. Preparation of the mRNA samples for sequencing was performed by ESALQ Genomics Center (Piracicaba, São Paulo, Brazil), using the TruSeq RNA Sample Preparation Kit^®^ (Illumina, San Diego, CA) according to manufacturer’s instructions. A description of the protocol was previously provided^12^. Cluster generation and sequencing were performed on the Illumina HiSeq 2000^®^. Paired-end reads of 2 × 100 bp were produced.

### Processing and alignment of sequence reads

Computations were performed on the HPC resources at the University of Missouri Bioinformatics Consortium (UMBC). Low-quality reads were filtered and adapter sequences trimmed using SeqClean software. The Tuxedo suite pipeline was used to perform the analysis as previously described^12^. TopHat v2.0.6^18^ was used to align the reads to the Bos taurus UMD3.1 reference genome.

### Transcript assembly and quantification

Transcript assembly and quantification was performed as described elsewhere^12^ using Cufflinks v2.0.2^22^,^23^, which was initially used to generate a reference annotation-based transcript assembly for each sample individually. The output for each sample included all reference transcripts as well as novel assembled genes and isoforms. Cufflinks assemblies for all samples were then merged using Cuffmerge v2.0.2. Cuffmerge v2.2.1 was then used to compare the assembled transcripts to reference transcripts.

### Testing for differential expression

Cuffdiff2 v2.2.1 was run to test for DE genes between the divergent RFI groups. The geometric normalization method and the pooled dispersion method were used to normalize the libraries and to model cross-replicate dispersion, respectively. Briefly, the Cuffdiff2 approach is to first model the variability in fragment count for each gene across biological replicates. The algorithm uses a beta negative binomial model to represent fragment count dispersion and to estimate count variances for each transcript, which are then used to perform a statistical test to identify significantly DE genes. A change in the expression level of a gene is measured by comparing its fragment count in each biological treatment^23^. Correction for multiple testing was performed using the Benjamini-Hochberg methodology. A false discovery rate ≤0.05 was adopted to consider a gene as being DE. Data exploration and visualization was performed using the CummeRbund package^22^ within the R programming environment. The DE gene matrix from Cuffdiff was used for principal component analyses.

### Annotation of differentially expressed unannotated transcripts

Sequences for the newly assembled transcripts were analyzed by BLAST to public database banks including SWISS-PROT (a manually annotated and reviewed protein sequence database), nr (NCBI non-redundant protein sequences), KEGG (Kyoto Encyclopedia of Genes and Genomes), KOG (euKaryotic Ortholog Groups), and Pfam (a widely used protein family and structure domain database).

### Functional annotation of differentially expressed known genes

We used the Database for Annotation, Visualization and Integrated Discovery (DAVID) v6.7^21^ to perform the functional annotation of the DE annotated gene lists. Annotation terms with EASE scores <0.1 (DAVID analysis) were considered significant. Finally, the GeneMANIA prediction server^24^ was used to visualize the biological network integration for the DE genes using human as the background.

## Additional Information

**Accession code:** The RNA-seq data sets supporting the results of this study are available in the ENA repository (EMBL-EBI), under study accession PRJEB15314.

**How to cite this article**: Tizioto, P. C. *et al*. Gene expression differences in *Longissimus* muscle of Nelore steers genetically divergent for residual feed intake. *Sci. Rep.* **6**, 39493; doi: 10.1038/srep39493 (2016).

**Publisher's note:** Springer Nature remains neutral with regard to jurisdictional claims in published maps and institutional affiliations.

## Supplementary Material

Supplementary Information

## Figures and Tables

**Table 1:** Best linear unbiased predictions of additive genetic merit for Residual Feed Intake (RFI); raw phenotypes for RFI, dry matter intake (DMI) and average daily gain (ADG); number of reads passing filtering and concordant pair alignment rate for each animal within the Low (LRFI, more efficient) or High (HRFI, less efficient) groups.

Animal_ID	Sample accession*	BLUP RFI (kg/day)	RFI (kg/day)	DMI (kg/day)	ADG (kg/day)	Reads passing filtering	Concordant pair alignment rate (%)
HRFI0	ERS1342435	0.125	1.808	10.53	1.39	20,238,310	92.10
HRFI1	ERS1342436	0.109	1.182	8.95	1.63	20,711,295	92.10
HRFI2	ERS1342437	0.094	0.659	10.07	1.54	17,055,887	91.90
HRFI3	ERS1342438	0.092	0.597	10.17	1.85	19,285,529	92.00
HRFI4	ERS1342439	0.090	0.496	8.54	1.54	18,410,456	92.10
HRFI5	ERS1342440	0.088	0.411	9.72	1.86	22,050,730	84.00
HRFI6	ERS1342441	0.087	0.421	10.32	1.92	19,119,265	91.60
HRFI7	ERS1342442	0.086	1.280	9.09	1.53	16,497,720	91.40
HRFI8	ERS1342443	0.086	0.327	8.97	1.66	14,395,852	91.60
HRFI9	ERS1342444	0.072	−0.155	9.78	1.78	19,750,013	92.40
mean		0.093	0.703	10.17	1.67	18,751,506	91.12
LRFI0	ERS1342445	−0.0990	−1.228	8.49	1.73	18,979,482	91.50
LRFI1	ERS1342446	−0.0914	−1.049	7.75	1.7	23,333,953	91.20
LRFI2	ERS1342447	−0.0862	−0.7682	8.57	1.75	16,798,528	90.00
LRFI3	ERS1342448	−0.0699	−0.5469	8.75	1.41	15,877,578	93.30
LRFI4	ERS1342449	−0.0341	−0.3803	8.38	1.39	22,993,080	80.50
LRFI5	ERS1342450	−0.0417	−0.1459	10.1	1.83	16,338,238	91.70
LRFI6	ERS1342451	−0.0414	−0.6588	7.43	0.98	16,332,732	91.70
LRFI7	ERS1342452	−0.0360	−0.5714	8.45	1.48	17,398,472	92.50
LRFI8	ERS1342453	−0.0679	−1.198	8.4	1.78	16,164,645	91.40
LRFI9	ERS1342454	−0.0306	−0.2845	10.41	2.33	15,321,005	91.40
mean		−0.0598	−0.6832	8.67	1.63	17,953,771	90.52

^*^Sample accession in the ENA repository (EMBL-EBI), under study accession PRJEB15314.

**Table 2:** Differentially expressed genes in the *Longissimus thoracis* muscle of high- and low- residual feed intake Nelore steers.

Gene symbol	Locus	FPKM mean in the LRFI, more efficient group	FPKM mean in the HRFI, less efficient group	FC	q-value
*ACSM1*	25:18,349,642–18,413,897	3.55	5.72	0.69	0.01
*ACTC1*	10:30,361,774–30,367,052	38.09	215.97	2.50	0.01
*AGTPBP1*	8:80,234,812–80,433,199	3.93	6.13	0.64	0.01
*ARID5B*	28:18,003,722–18,195,950	8.95	14.85	0.73	0.01
*ARRDC3*	7:93,238,461–93,253,182	11.18	17.37	0.64	0.01
*ATF3*	16:72,819,913–72,878,316	37.33	69.89	0.90	0.01
*B3GALTL*	12:29,659,763–29,776,394	2.58	469.34	7.51	0.01
*BDH1*	1:72,572,762–72,610,397	1.81	4.29	1.25	0.01
*BLA-DQB*	23:25,855,144–25,863,045	18.93	11.68	−0.70	0.01
*BTG2*	16:889,144–894,202	28.67	39.98	0.48	0.04
*C3*	7:18,989,626–19,025,593	1.08	0.38	−1.51	0.01
*CASQ2*	3:27,658,847–27,729,891	18.89	26.59	0.49	0.03
*CD109*	9:13,421,006–13,556,277	3.24	4.63	0.52	0.01
*CD209*	7:17,810,225–17,814,276	5.53	9.50	0.78	0.03
*CHRND*	2:120,974,969–120,982,375	3.81	9.52	1.32	0.01
*CISH*	22:50,320,098–50,325,618	9.66	6.026	−0.68	0.01
*COL14A1*	14:83,876,538–84,110,654	8.21	11.82	0.53	0.01
*COL6A6*	1:153,321,010–153,494,877	1.80	2.74	0.61	0.01
*COQ10B*	2:86,410,716–86,427,939	10.36	17.46	0.75	0.01
*CYP1A1*	21:34,340,289–34,351,607	8.57	5.79	−0.57	0.01
*CYP1B1*	11:20,490,140–20,499,187	0.73	1.42	0.95	0.04
*EBF1*	7:72,393,224–72,804,822	5.59	7.78	0.48	0.04
*EFEMP1*	11:38,338,736–38,408,331	14.28	19.66	0.46	0.03
*EGR1*	7:51,438,703–51,442,530	23.47	39.95	0.77	0.01
*EIF4EBP1*	27:32,951,593–32,973,439	60.84	43.39	−0.49	0.05
*EMILIN2*	24:37,496,209–37,556,462	2.80	4.07	0.54	0.02
*ENAH*	16:29,229,604–29,442,778	8.41	12.62	0.59	0.01
*F13A1*	23:48,633,935–48,776,698	6.80	9.75	0.52	0.01
*FSTL1*	1:65,742,625–65,802,423	53.10	77.77	0.55	0.04
*GAS7*	19:29,665,966–29,876,650	3.17	4.67	0.56	0.02
*GFPT2*	7:772,140–817,776	5.34	8.00	0.58	0.01
*GSTP1*	29:46,087,141–46,090,009	99.65	72.21	−0.46	0.02
*HOXC10*	5:26,201,346–26,215,613	13.95	9.94	−0.49	0.04
*IER5*	16:63,677,268–63,691,626	12.94	18.76	0.54	0.01
*IFI6*	2:126,246,575–126,250,174	11.55	19.93	0.79	0.03
*KERA*	5:20,995,310–21,003,085	1.96	3.87	0.98	0.01
*KIAA1551*	5:78,299,287–78,326,541	2.73	3.79	0.48	0.05
*LIMK1*	25:33,749,961–33,775,754	4.61	3.022	−0.60	0.04
*LOC100300305*	23:30,817,650–30,825,629	8.59	15.45	0.85	0.01
*LOC100335754*	11:104,181,959–104,185,687	153.48	90.65	−0.76	0.01
*LOC100336823*	29:44,770,858–44,771,621	75.35	45.50	−0.73	0.01
*LOC100848726*	29:50,712,831–50,713,218	7453.97	151.88	−5.62	0.01
*LOC100848909*	6:92,504,246–92,544,085	172.60	0.52	−8.39	0.01
*LOC782776*	1:77,035,495–77,037,254	25.62	45.29	0.82	0.01
*LOC787803*	X:94,701,246–94,701,909	11.88	1.97	−2.59	0.01
*LYVE1*	15:42,678,143–42,693,086	6.48	9.93	0.62	0.01
*MDM4*	16:2,065,963–2,106,826	3.82	5.62	0.56	0.05
*MEDAG*	12:30,016,965–30,040,051	12.83	17.77	0.47	0.04
*MPZ*	3:8,229,125–8,235,252	19.54	12.56	−0.64	0.01
*MYC*	14:13,769,241–13,774,939	5.61	8.55	0.61	0.02
*MYL3*	22:53,161,299–53,224,114	790.03	377.60	−1.07	0.01
*NDUFS7*	7:45,427,303–45,433,438	284.04	199.60	−0.51	0.04
*NIN*	10:43,637,003–43,745,262	1.74	2.46	0.50	0.04
*NOV*	14:47,005,556–47,014,101	14.72	23.45	0.67	0.01
*NR4A2*	2:39,944,984–40,017,777	4.94	8.51	0.78	0.01
*OTUD1*	13:24,655,213–24,658,469	104.97	172.28	0.71	0.02
*PARP2*	10:26,799,838–26,814,591	9.40	5.11	−0.88	0.01
*PI15*	14:40,275,630–40,313,055	0.94	2.13	1.18	0.01
*PKHD1L1*	14:57,040,806–57,216,685	0.43	0.76	0.84	0.01
*RIF1*	2:44,756,972–44,819,250	3.36	4.66	0.47	0.01
*RN18S1*	25:32,428,075–32,430,097	20102.70	835.18	−4.59	0.01
*RN28S1*	3:53,470,477–53,472,889	8751.13	351.13	−4.64	0.01
*RN28S1*	3:35,428,057–35,862,958	9997.71	376.81	−4.73	0.01
*RN5-8S1*	25:32,467,531–32,467,688	32282.90	293.23	−6.78	0.01
*RSPO2*	14:58,537,058–58,709,246	0.29	1.51	2.38	0.01
*SBK2*	18:62,434,331–62,439,412	0.83	3.22	1.95	0.01
*SLC26A2*	7:63,280,898–63,303,387	0.64	1.09	0.77	0.01
*TGFBR3*	3:51,660,730–51,870,069	13.65	19.77	0.53	0.05
*THBS3*	3:15,470,267–15,480,514	1.60	2.73	0.77	0.02
*TIPARP*	1:111,829,353–111,857,046	4.79	7.58	0.66	0.01
*TNMD*	X:50,951,554–50,973,156	1.75	5.10	1.54	0.01
*UCHL1*	6:61,785,263–61,807,737	9.82	5.97	−0.72	0.01
*WDR74*	29:41,838,790–41,846,756	13.40	8.95	−0.58	0.01

LRFI = Low Residual Feed Intake; HRFI = High Residual Feed Intake; FC = log_2_-fold change; q-value = FDR-adjusted p-value.

**Table 3:** Summary of Biological Processes from the Gene Ontology provided by DAVID.

Term	P-Value	Genes
GO:0010033~response to organic substance	0.02	*EGR1, EIF4EBP1, NR4A2, MYC*
GO:0001836~release of cytochrome c from mitochondria	0.03	*MYC, IFI6*
GO:0008637~apoptotic mitochondrial changes	0.04	*MYC, IFI6*
GO:0043066~negative regulation of apoptosis	0.07	*NR4A2, MYC, IFI6*
GO:0008344~adult locomotory behavior	0.07	*UCHL1, NR4A2*
GO:0043069~negative regulation of programmed cell death	0.07	*NR4A2, MYC, IFI6*
GO:0060548~negative regulation of cell death	0.07	*NR4A2, MYC, IFI6*
GO:0043085~positive regulation of catalytic activity	0.08	*NR4A2, MYC, CISH*
GO:0009057~macromolecule catabolic process	0.08	*LYVE1, UCHL1, MYC, CISH*
GO:0043281~regulation of caspase activity	0.09	*MYC, IFI6*
GO:0052548~regulation of endopeptidase activity	0.10	*MYC, IFI6*
GO:0044093~positive regulation of molecular function	0.10	*NR4A2, MYC, CISH*
