# Corrigendum: Gene expression differences in *Longissimus* muscle of Nelore steers genetically divergent for residual feed intake

**DOI:** 10.1038/srep44345

**Published:** 2017-04-05

**Authors:** Polyana C. Tizioto, Luiz L. Coutinho, Priscila S. N. Oliveira, Aline S. M. Cesar, Wellison J. S. Diniz, Andressa O. Lima, Marina I. Rocha, Jared E. Decker, Robert D. Schnabel, Gerson B. Mourão, Rymer R. Tullio, Adhemar Zerlotini, Jeremy F. Taylor, Luciana C. A. Regitano

Scientific Reports 6: Article number: 3949310.1038/srep39493; published online: 12 22 2016; updated: 04 05 2017

This Article contains an error in the order of the Figures, where Figures 2 and 3 were inverted. The correct Figures 2 and 3 appear below:

## Figures and Tables

**Figure 2:**
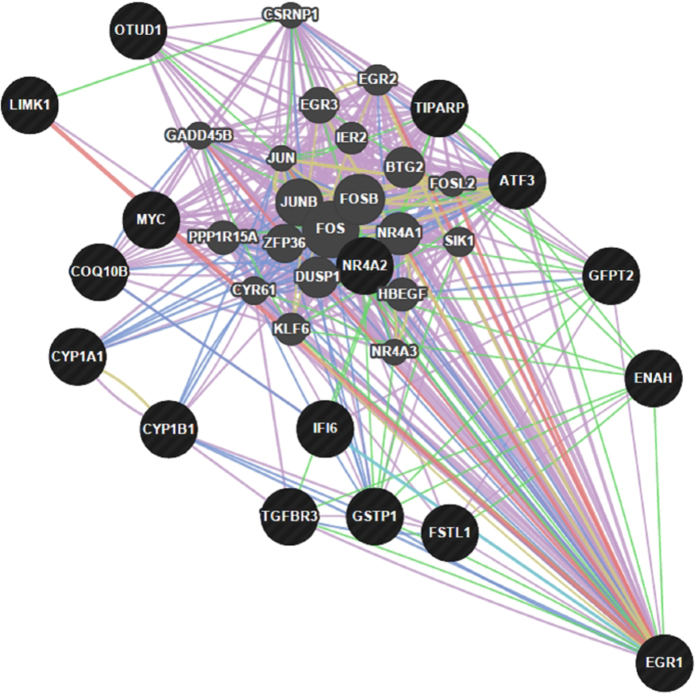
Network integration of the EGR1 gene and other differentially expressed genes. Genes presented as black circles were differentially expressed (DE) between the efficient (LRFI) and inefficient (HRFI) groups. Genes presented in grey interact with the DE genes. Arrows presented in pink, green, blue and red represent coexpression relationships, genetic interactions, co-localizations and physical interactions, respectively.

**Figure 3:**
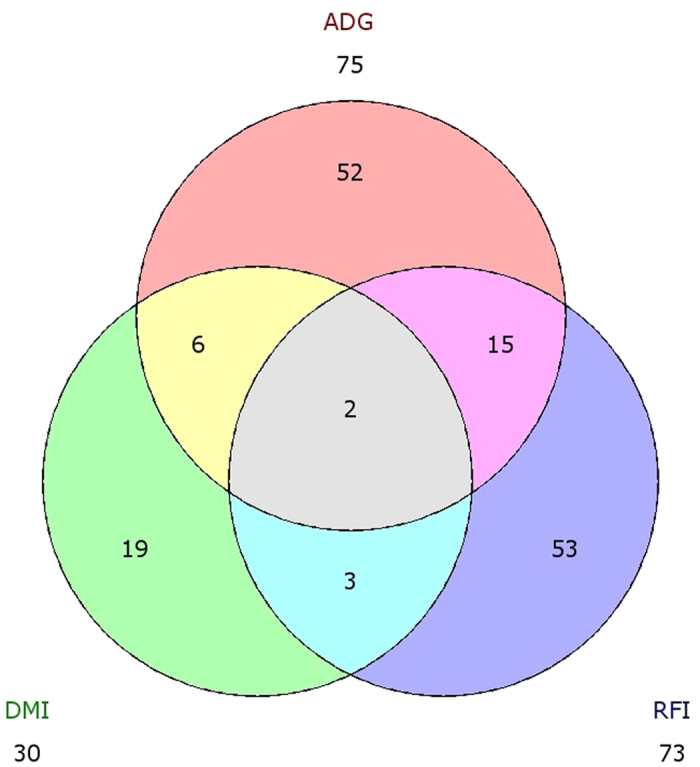
Venn diagram showing overlaps between differentially expressed genes found for residual feed intake (RFI), average daily gain (ADG) and dry matter intake (DMI).

