# Supplementary material for: Gene expression differences in Longissimus muscle of Nelore steers genetically divergent for residual feed intake
Source: Sci Rep. 2016 Dec 22;6:39493. doi: 10.1038/srep39493 (PMC5177880; doi:10.1038/srep39493)
Supplement: Supplementary Information [file srep39493-s1.doc]

**Gene expression differences in *Longissimus* muscle of Nelore steers genetically divergent for residual feed intake**

Polyana C Tizioto^1,2^, Luiz L Coutinho^3^, Priscila SN Oliveira^1^, Aline SM Cesar^3^, Wellison JS Diniz^4^, Andressa O Lima^4^, Marina I Rocha^4^, Jared E Decker^2,5^, Robert D Schnabel^2,5^, Gerson B Mourão^3^, Rymer R Tullio^1^, Adhemar Zerlotini^6^, Jeremy F Taylor^2^ & Luciana CA Regitano^1*^

^1^ Embrapa Pecuária Sudeste, São Carlos, SP, Brazil

^2^ Division of Animal Sciences, University of Missouri Columbia, Columbia, MO, USA

^3^ Department of Animal Science, University of São Paulo/ESALQ, Piracicaba, São Paulo, Brazil

^4^ Department of Genetics and Evolution, Federal University of São Carlos, São Carlos, SP, Brazil

^5^Informatics Institute, University of Missouri, Columbia, Missouri 65211, USA

^6^ Embrapa Informática Agropecuária, Campinas, SP, Brazil

^*^Corresponding author: luciana.regitano@embrapa.br

**Supplementary material**

**Supplementary Table S1. Annotation results by BLAST of unknown differentially expressed transcripts between the more efficient (LRFI) and less efficient (HRFI) groups.**

| Gene | Locus | LRFI, more efficient | HRFI, less efficient | FC | q_value | Alignment | E value | Identity (%) |
| --- | --- | --- | --- | --- | --- | --- | --- | --- |
| Unknown1 | 2:66269150-66269393 | 36.78 | 1.73 | -4.41 | 0.01 | PREDICTED: Bos taurus uncharacterized LOC107132266 (LOC107132266), ncRNA | 1.0E-63 | 100 |
| Unknown2 | 3:12281795-12289777 | 1.05 | 0.53 | -0.99 | 0.04 | PREDICTED: Bos taurus uncharacterized LOC104971427 (LOC104971427), ncRNA | 0.0 | 99 |
| Unknown 3 | 12:83120272-83124234 | 0.47 | 0.96 | 1.02 | 0.05 | Bos taurus T cell receptor gamma cluster 1 (TCRG1) gene, complete sequence | 0.0 | 94 |
| Unknown 4 | 19:6012648417-60651311 | 0.61 | 2.45 | 2.00 | 0.01 | PREDICTED: Bos taurus uncharacterized LOC104975158 (LOC104975158), ncRNA | 2.0E-17 | 100 |
| Unknown5 | 20:5396337-5401090 | 0.83 | 1.94 | 1.22 | 0.01 | PREDICTED: Bos taurus uncharacterized LOC104976640 (LOC104976640), transcript variant X2, ncRNA | 0.0 | 100 |
| Unknown6 | 23:24977637-24977974 | 149.58 | 2.59 | -5.85 | 0.01 | Homo sapiens RNA, 7SK small nuclear (RN7SK), small nuclear RNA | 6.0E-17 | 99 |
| Unknown7 | 23:6106218-6117781 | 0.55 | 0.87 | 0.67 | 0.01 | Bos taurus Y Chr BAC CH240-259D2 complete sequence | 0.0 | 86 |
| Unknown8 | 25:32373864-32374138 | 28.05 | 10.94 | -1.36 | 0.02 | PREDICTED: Bos taurus basic salivary proline-rich protein 4-like (LOC101909230), transcript variant X1, mRNA | 9.0E-59 | 99 |
| Unknown9 | 25:32406822-32406991 | 120.69 | 5.33 | -4.50 | 0.01 | Sus scrofa 18S ribosomal RNA (RN18S), ribosomal RNA | 6.0E-78 | 99 |
| Unknown10 | 25:32458798-32459031 | 25.11 | 1.91 | -3.72 | 0.01 | Sus scrofa 18S ribosomal RNA (RN18S), ribosomal RNA | 1.0E-11 | 100 |
| Unknown11 | X:114170885-114171187 | 283.71 | 182.29 | -0.64 | 0.04 | Bos grunniens isolate Gobi42 D-loop, partial sequence; mitochondrial | 7E-14 | 98 |

LRFI= Low Residual Feed Intake; HRFI= High Residual Feed Intake; FC= log_2_-fold change; q-value= FDR-adjusted p-value; E-value= Expect value

**Supplementary Table S2. List of functional clusters found for differentially expressed genes between the more efficient (LRFI) and less efficient (HRFI) groups.**

| Annotation Cluster 1 Enrichment Score: 1.20 | | | |
| --- | --- | --- | --- |
| Category | Term | Genes | Pvalue |
| GOTERM_MF_FAT | GO:0005509~calcium ion binding | MYL3, F13A1, EFEMP1, FSTL1, THBS3, CASQ2 | 0.03 |
| GOTERM_MF_FAT | GO:0046872~metal ion binding | EGR1, CYP1A1, MYL3, AGTPBP1, F13A1, TIPARP, EFEMP1, NR4A2, FSTL1, NDUFS7, ACSM1, MDM4, THBS3, CASQ2 | 0.04 |
| GOTERM_MF_FAT | GO:0043169~cation binding | EGR1, CYP1A1, MYL3, AGTPBP1, F13A1, TIPARP, EFEMP1, NR4A2, FSTL1, NDUFS7, ACSM1, MDM4, THBS3, CASQ2 | 0.04 |
| GOTERM_MF_FAT | GO:0043167~ion binding | EGR1, CYP1A1, MYL3, AGTPBP1, F13A1, TIPARP, EFEMP1, NR4A2, FSTL1, NDUFS7, ACSM1, MDM4, THBS3, CASQ2 | 0.04 |
| GOTERM_MF_FAT | GO:0046914~transition metal ion binding | NDUFS7, EGR1, CYP1A1, AGTPBP1, TIPARP, NR4A2, MDM4 | 0.52 |
|  |  |  |  |
| Annotation Cluster 2 Enrichment Score: 0.88 | | | |
| Category | Term | Genes | Pvalue |
| GOTERM_BP_FAT | GO:0043066~negative regulation of apoptosis | NR4A2, MYC, IFI6 | 0.07 |
| GOTERM_BP_FAT | GO:0060548~negative regulation of cell death | NR4A2, MYC, IFI6 | 0.07 |
| GOTERM_BP_FAT | GO:0043069~negative regulation of programmed cell death | NR4A2, MYC, IFI6 | 0.07 |
| GOTERM_BP_FAT | GO:0016265~death | NR4A2, MYC, IFI6 | 0.14 |
| GOTERM_BP_FAT | GO:0042981~regulation of apoptosis | NR4A2, MYC, IFI6 | 0.23 |
| GOTERM_BP_FAT | GO:0043067~regulation of programmed cell death | NR4A2, MYC, IFI6 | 0.23 |
| GOTERM_BP_FAT | GO:0010941~regulation of cell death | NR4A2, MYC, IFI6 | 0.23 |
|  |  |  |  |
| Annotation Cluster 3 Enrichment Score: 0.61 | | | |
| Category | Term | Genes | Pvalue |
| GOTERM_BP_FAT | GO:0010033~response to organic substance | EGR1, EIF4EBP1, NR4A2, MYC | 0.02 |
| GOTERM_BP_FAT | GO:0006350~transcription | EGR1, ATF3, NR4A2, MYC | 0.19 |
| GOTERM_MF_FAT | GO:0003700~transcription factor activity | EGR1, ATF3, NR4A2, MYC | 0.19 |
| GOTERM_MF_FAT | GO:0030528~transcription regulator activity | EGR1, ATF3, EBF1, NR4A2, MYC | 0.21 |
| GOTERM_BP_FAT | GO:0006355~regulation of transcription, DNA-dependent | EGR1, ATF3, EBF1, NR4A2, MYC | 0.26 |
| SP_PIR_KEYWORDS | transcription regulation | EGR1, ATF3, NR4A2, MYC | 0.26 |
| GOTERM_BP_FAT | GO:0051252~regulation of RNA metabolic process | EGR1, ATF3, EBF1, NR4A2, MYC | 0.27 |
| SP_PIR_KEYWORDS | dna-binding | EGR1, ATF3, NR4A2, MYC | 0.29 |
| SP_PIR_KEYWORDS | Transcription | EGR1, ATF3, NR4A2, MYC | 0.34 |
| GOTERM_MF_FAT | GO:0003677~DNA binding | EGR1, ATF3, EBF1, NR4A2, MYC | 0.47 |
| GOTERM_BP_FAT | GO:0045449~regulation of transcription | EGR1, ATF3, EBF1, NR4A2, MYC | 0.49 |
| SP_PIR_KEYWORDS | nucleus | EGR1, WDR74, ATF3, NR4A2, MDM4, MYC | 0.67 |
|  |  |  |  |
| Annotation Cluster 4 Enrichment Score: 0.46 | | | |
| Category | Term | Genes | Pvalue |
| GOTERM_CC_FAT | GO:0070013~intracellular organelle lumen | WDR74, ACSM1, ATF3, MYC, BDH1 | 0.17 |
| GOTERM_CC_FAT | GO:0043233~organelle lumen | WDR74, ACSM1, ATF3, MYC, BDH1 | 0.17 |
| GOTERM_CC_FAT | GO:0031974~membrane-enclosed lumen | WDR74, ACSM1, ATF3, MYC, BDH1 | 0.18 |
| GOTERM_CC_FAT | GO:0043228~non-membrane-bounded organelle | WDR74, ACTC1, ATF3, MYL3, LOC787803, MYC | 0.43 |
| GOTERM_CC_FAT | GO:0043232~intracellular non-membrane-bounded organelle | WDR74, ACTC1, ATF3, MYL3, LOC787803, MYC | 0.43 |
| GOTERM_CC_FAT | GO:0031981~nuclear lumen | WDR74, ATF3, MYC | 0.48 |
| GOTERM_CC_FAT | GO:0005856~cytoskeleton | ACTC1, MYL3, MYC | 0.64 |
| SP_PIR_KEYWORDS | nucleus | EGR1, WDR74, ATF3, NR4A2, MDM4, MYC | 0.67 |
|  |  |  |  |
| Annotation Cluster 5 Enrichment Score: 0.44 | | | |
| Category | Term | Genes | Pvalue |
| SP_PIR_KEYWORDS | glycoprotein | LYVE1, MPZ, KERA, C3, BLA-DQB, UCHL1, FSTL1, CHRND, MYC, SLC26A2 | 0.03 |
| SP_PIR_KEYWORDS | signal | LYVE1, MPZ, KERA, C3, BLA-DQB, FSTL1, CHRND, IFI6 | 0.11 |
| SP_PIR_KEYWORDS | disulfide bond | LYVE1, MPZ, KERA, C3, BLA-DQB, FSTL1, CHRND | 0.13 |
| UP_SEQ_FEATURE | disulfide bond | LYVE1, MPZ, KERA, C3, BLA-DQB, FSTL1, CHRND | 0.17 |
| UP_SEQ_FEATURE | signal peptide | LYVE1, MPZ, KERA, C3, BLA-DQB, FSTL1, CHRND, IFI6 | 0.19 |
| UP_SEQ_FEATURE | topological domain:Extracellular | LYVE1, MPZ, BLA-DQB, CHRND, SLC26A2 | 0.26 |
| UP_SEQ_FEATURE | glycosylation site:N-linked (GlcNAc...) | LYVE1, KERA, C3, BLA-DQB, FSTL1, CHRND, SLC26A2 | 0.34 |
| SP_PIR_KEYWORDS | Secreted | KERA, C3, F13A1, FSTL1 | 0.47 |
| UP_SEQ_FEATURE | topological domain:Cytoplasmic | LYVE1, MPZ, BLA-DQB, CHRND, SLC26A2 | 0.51 |
| SP_PIR_KEYWORDS | transport | NDUFS7, LYVE1, CHRND, SLC26A2 | 0.64 |
| SP_PIR_KEYWORDS | transmembrane | LYVE1, MPZ, BLA-DQB, CD209, CHRND, SLC26A2, IFI6 | 0.75 |
| UP_SEQ_FEATURE | transmembrane region | LYVE1, MPZ, BLA-DQB, CHRND, SLC26A2, IFI6 | 0.77 |
| GOTERM_CC_FAT | GO:0005886~plasma membrane | LYVE1, BLA-DQB, CHRND, CISH | 0.84 |
| SP_PIR_KEYWORDS | membrane | LYVE1, MPZ, BLA-DQB, CD209, CHRND, SLC26A2, IFI6 | 0.89 |
| GOTERM_CC_FAT | GO:0016021~integral to membrane | LYVE1, MPZ, BLA-DQB, CD209, CHRND, SLC26A2, IFI6 | 0.99 |
| GOTERM_CC_FAT | GO:0031224~intrinsic to membrane | LYVE1, MPZ, BLA-DQB, CD209, CHRND, SLC26A2, IFI6 | 0.99 |
|  |  |  |  |
| Annotation Cluster 6 Enrichment Score: 0.32 | | | |
| Category | Term | Genes | Pvalue |
| SP_PIR_KEYWORDS | transit peptide | NDUFS7, ACSM1, BDH1 | 0.38 |
| GOTERM_CC_FAT | GO:0044429~mitochondrial part | NDUFS7, ACSM1, BDH1 | 0.42 |
| UP_SEQ_FEATURE | transit peptide:Mitochondrion | NDUFS7, ACSM1, BDH1 | 0.44 |
| GOTERM_CC_FAT | GO:0005739~mitochondrion | NDUFS7, ACSM1, BDH1, IFI6 | 0.52 |
| SP_PIR_KEYWORDS | mitochondrion | NDUFS7, ACSM1, BDH1 | 0.66 |
|  |  |  |  |
| Annotation Cluster 7 Enrichment Score: 0.21 | | | |
| Category | Term | Genes | Pvalue |
| SP_PIR_KEYWORDS | zinc-finger | EGR1, NR4A2, MDM4 | 0.47 |
| GOTERM_MF_FAT | GO:0046914~transition metal ion binding | NDUFS7, EGR1, CYP1A1, AGTPBP1, TIPARP, NR4A2, MDM4 | 0.52 |
| SP_PIR_KEYWORDS | metal-binding | NDUFS7, EGR1, ACSM1, NR4A2, MDM4 | 0.66 |
| SP_PIR_KEYWORDS | nucleus | EGR1, WDR74, ATF3, NR4A2, MDM4, MYC | 0.67 |
| GOTERM_MF_FAT | GO:0008270~zinc ion binding | EGR1, AGTPBP1, TIPARP, NR4A2, MDM4 | 0.69 |
| SP_PIR_KEYWORDS | zinc | EGR1, NR4A2, MDM4 | 0.80 |
|  |  |  |  |
| Annotation Cluster 8 Enrichment Score: 0.06 | | | |
| Category | Term | Genes | Pvalue |
| GOTERM_MF_FAT | GO:0032553~ribonucleotide binding | LOC782776, ACTC1, ACSM1, SBK2 | 0.83 |
| GOTERM_MF_FAT | GO:0032555~purine ribonucleotide binding | LOC782776, ACTC1, ACSM1, SBK2 | 0.83 |
| SP_PIR_KEYWORDS | nucleotide-binding | LOC782776, ACTC1, ACSM1 | 0.85 |
| GOTERM_MF_FAT | GO:0017076~purine nucleotide binding | LOC782776, ACTC1, ACSM1, SBK2 | 0.86 |
| GOTERM_MF_FAT | GO:0005524~ATP binding | ACTC1, ACSM1, SBK2 | 0.87 |
| GOTERM_MF_FAT | GO:0032559~adenyl ribonucleotide binding | ACTC1, ACSM1, SBK2 | 0.87 |
| GOTERM_MF_FAT | GO:0030554~adenyl nucleotide binding | ACTC1, ACSM1, SBK2 | 0.89 |
| GOTERM_MF_FAT | GO:0001883~purine nucleoside binding | ACTC1, ACSM1, SBK2 | 0.90 |
| GOTERM_MF_FAT | GO:0001882~nucleoside binding | ACTC1, ACSM1, SBK2 | 0.90 |
| GOTERM_MF_FAT | GO:0000166~nucleotide binding | LOC782776, ACTC1, ACSM1, SBK2 | 0.93 |
|  |  |  |  |

**Supplementary Table S3. List of differentially expressed genes for average daily gain (ADG) between the divergent residual feed intake (RFI) groups**

| Gene Symbol | Locus | Low-ADG | High-ADG | FC | q-value |
| --- | --- | --- | --- | --- | --- |
| ACTC1 | 10:30361774-30367052 | 37.97 | 82.84 | 1.13 | 0.01 |
| ADIPOQ | 1:81005164-81018531 | 20.18 | 45.19 | 1.16 | 0.01 |
| ANGPTL1 | 16:61559976-61737959 | 7.50 | 13.27 | 0.82 | 0.03 |
| ANGPTL5 | 15:7148660-7165793 | 0.50 | 1.73 | 1.80 | 0.02 |
| ATF3 | 16:72819913-72878316 | 32.48 | 67.42 | 1.05 | 0.04 |
| BRB | 10:26288222-26289852 | 21.77 | 34.17 | 0.65 | 0.01 |
| MEDAG | 12:30016965-30040051 | 11.85 | 19.51 | 0.72 | 0.01 |
| CCDC3 | 13:11512165-11636251 | 6.90 | 11.86 | 0.78 | 0.01 |
| CD55 | 16:5099074-5124229 | 2.44 | 4.24 | 0.80 | 0.04 |
| CDO1 | 10:4598429-4606919 | 2.73 | 5.59 | 1.04 | 0.02 |
| COL22A1 | 14:5131801-5354761 | 0.47 | 1.20 | 1.36 | 0.01 |
| COL6A6 | 1:153321010-153494877 | 1.76 | 2.62 | 0.57 | 0.04 |
| COL8A1 | 1:43541776-43717682 | 3.53 | 6.37 | 0.85 | 0.01 |
| CRISPLD2 | 18:10985131-11050908 | 19.39 | 29.86 | 0.62 | 0.01 |
| CTHRC1 | 14:63311959-63328221 | 1.35 | 3.46 | 1.35 | 0.03 |
| CYP1B1 | 11:20490140-20499187 | 0.49 | 1.63 | 1.72 | 0.01 |
| CYP26B1 | 11:12370497-12390070 | 1.51 | 3.39 | 1.17 | 0.01 |
| DCLK1 | 12:25362925-25718173 | 1.50 | 2.74 | 0.87 | 0.02 |
| EFEMP1 | 11:38338736-38408331 | 13.95 | 21.15 | 0.60 | 0.01 |
| FABP4 | 14:46833664-46838053 | 26.38 | 68.73 | 1.38 | 0.01 |
| FBLN7 | 11:188323-254340 | 1.51 | 4.84 | 1.67 | 0.01 |
| FMOD | 16:925080-935146 | 7.78 | 22.10 | 1.51 | 0.01 |
| FOS | 10:86883738-86887170 | 94.53 | 148.03 | 0.65 | 0.01 |
| FOSL2 | 11:71326072-71349903 | 8.37 | 13.65 | 0.70 | 0.01 |
| FOXO1 | 12:21915283-22009120 | 22.05 | 35.06 | 0.67 | 0.01 |
| FSTL1 | 1:65742625-65802423 | 47.86 | 82.44 | 0.78 | 0.01 |
| GALNTL2 | 1:154922559-154969506 | 1.71 | 3.83 | 1.16 | 0.01 |
| GLCE | 10:16009393-16119073 | 4.52 | 9.07 | 1.00 | 0.01 |
| IER5 | 16:63677268-63691626 | 11.96 | 19.10 | 0.68 | 0.01 |
| ITGBL1 | 12:81873526-82108998 | 8.64 | 15.38 | 0.83 | 0.01 |
| LHFP | 12:22817611-23053616 | 10.23 | 15.16 | 0.57 | 0.03 |
| LOC100335754 | 11:104181959-104185687 | 139.93 | 86.17 | -0.70 | 0.01 |
| LOC100337053 | 12:72006072-72205713 | 2.00 | 1.05 | -0.92 | 0.03 |
| LOC100848491 | 11:106854026-106946517 | 16.21 | 24.76 | 0.61 | 0.01 |
| LOC100848684 | 1:84252018-84259770 | 4.11 | 1.45 | -1.50 | 0.01 |
| LOC504773 | 19:14730634-14741999 | 18.37 | 31.11 | 0.76 | 0.01 |
| PARP2 | 10:26799838-26814591 | 13.78 | 4.90 | -1.49 | 0.01 |
| LOC515150 | 16:4998255-5006453 | 11.78 | 20.44 | 0.79 | 0.01 |
| COL14A1 | 14:83876538-84110654 | 8.17 | 12.58 | 0.62 | 0.01 |
| LOC782776 | 1:77035495-77037254 | 25.44 | 43.80 | 0.78 | 0.01 |
| LOC786073 | 11:107260658-107267533 | 6.24 | 10.51 | 0.75 | 0.02 |
| LYVE1 | 15:42678143-42693086 | 5.50 | 11.05 | 1.01 | 0.01 |
| MATN2 | 14:68478150-68651754 | 4.06 | 6.24 | 0.62 | 0.02 |
| METTL11B | 16:38567908-38588018 | 6.66 | 3.99 | -0.74 | 0.04 |
| MMP14 | 10:21804500-21814925 | 6.55 | 9.64 | 0.56 | 0.04 |
| MMP16 | 14:76757536-77132857 | 1.98 | 4.05 | 1.03 | 0.01 |
| MX1 | 1:143172650-143205055 | 2.88 | 5.16 | 0.84 | 0.01 |
| MYC | 14:13769241-13774939 | 4.31 | 8.29 | 0.94 | 0.01 |
| MYH13 | 19:29956265-30008127 | 0.44 | 1.56 | 1.82 | 0.01 |
| MYH8 | 19:30031026-30061053 | 9.19 | 19.08 | 1.05 | 0.01 |
| MYLK3 | 18:15086118-15143252 | 6.51 | 3.68 | -0.82 | 0.01 |
| NOV | 14:47005556-47014101 | 12.98 | 22.90 | 0.82 | 0.01 |
| OAS1 | 17:63653010-63660161 | 2.10 | 4.23 | 1.01 | 0.03 |
| OTUD1 | 13:24655213-24658469 | 107.49 | 180.64 | 0.75 | 0.03 |
| P4HB | 19:51643809-51653754 | 46.57 | 67.94 | 0.54 | 0.04 |
| PAMR1 | 15:66754733-66832246 | 0.39 | 0.99 | 1.34 | 0.05 |
| PCOLCE2 | 1:127112669-127229654 | 29.88 | 46.13 | 0.63 | 0.01 |
| PI15 | 14:40275630-40313055 | 0.98 | 2.29 | 1.23 | 0.01 |
| POSTN | 12:24241673-24276725 | 2.28 | 4.54 | 0.99 | 0.01 |
| PPP1R15A | 18:55925612-55929301 | 26.33 | 39.09 | 0.57 | 0.02 |
| PROCR | 13:65051564-65125014 | 12.09 | 22.38 | 0.89 | 0.01 |
| PTGIS | 13:78315823-78368791 | 8.36 | 13.21 | 0.66 | 0.01 |
| RAMP2 | 19:43441582-43443429 | 45.25 | 66.31 | 0.55 | 0.03 |
| RCAN1 | 1:242291-362919 | 41.18 | 84.43 | 1.04 | 0.02 |
| RCN1 | 15:63656457-63664372 | 26.43 | 38.68 | 0.55 | 0.02 |
| ROCK2 | 11:86501574-86587468 | 20.82 | 32.47 | 0.64 | 0.04 |
| SDC4 | 13:74391485-74412874 | 17.11 | 33.28 | 0.96 | 0.01 |
| SERPINH1 | 15:55514858-55525178 | 42.28 | 63.26 | 0.58 | 0.02 |
| THBD | 13:42217370-42221004 | 9.46 | 15.15 | 0.68 | 0.01 |
| THBS1 | 10:35313982-35329775 | 12.51 | 21.53 | 0.78 | 0.01 |
| THBS4 | 10:10945038-10999249 | 35.58 | 94.25 | 1.41 | 0.02 |
| THY1 | 15:30509503-30515399 | 17.60 | 32.13 | 0.87 | 0.01 |
| TMEM119 | 17:66586627-66593119 | 0.51 | 1.42 | 1.47 | 0.04 |
| TUSC5 | 19:22167560-22186258 | 1.82 | 6.92 | 1.93 | 0.01 |
| ULBP3 | 17:39881233-39884975 | 16.24 | 7.18 | -1.18 | 0.01 |

Low-ADG= Low Average Daily Gain; High-ADG= High Average Daily Gain; FC= log_2_-fold change;

q-value= FDR-adjusted p-value.

**Supplementary Table S4. List of differentially expressed genes for dry matter intake (DMI) between the divergent residual feed intake (RFI) groups**

| Gene symbol | Locus | Low-DMI | High-DMI | FC | q-value |
| --- | --- | --- | --- | --- | --- |
| ACTC1 | 10:30361774-30367052 | 15.42 | 35.27 | 1.19 | 0.02 |
| APEX1 | 10:26706593-26709174 | 14.09 | 29.40 | 1.06 | 0.02 |
| BLA-DQB | 23:25855144-25863045 | 2.71 | 7.74 | 1.51 | 0.02 |
| BRB | 10:26288222-26289852 | 9.25 | 28.79 | 1.64 | 0.02 |
| C10H14orf166 | 10:44879975-44894548 | 54.93 | 122.08 | 1.15 | 0.02 |
| COMP | 7:4354382-4362113 | 2.64 | 0.69 | -1.93 | 0.02 |
| ERH | 10:81520783-81533472 | 21.86 | 44.69 | 1.03 | 0.02 |
| FABP4 | 14:46833664-46838053 | 23.09 | 47.88 | 1.05 | 0.02 |
| FOS | 10:86883738-86887170 | 50.96 | 118.79 | 1.22 | 0.02 |
| HSPA6 | 3:8027759-8029969 | 7.26 | 2.89 | -1.33 | 0.02 |
| LGALS3 | 10:67843327-67861114 | 5.18 | 13.30 | 1.36 | 0.02 |
| LOC100300267 | 6:41169070-41171884 | 0 | 2.75 | - | 0.02 |
| LOC100300305 | 23:30817650-30825629 | 1.89 | 7.12 | 1.91 | 0.02 |
| LOC100848726 | 29:50712831-50713218 | 222.64 | 49.77 | -2.16 | 0.02 |
| LOC100848979 | 10:61438749-61445849 | 2.81 | 10.44 | 1.89 | 0.02 |
| LOC782776 | 1:77035495-77037254 | 16.99 | 41.13 | 1.28 | 0.02 |
| LOC789192 | 25:27618-33338 | 8.13 | 26.12 | 1.68 | 0.02 |
| MYH13 | 19:29956265-30008127 | 0.42 | 1.17 | 1.47 | 0.02 |
| MYH8 | 19:30031026-30061053 | 4.02 | 10.30 | 1.36 | 0.02 |
| NEDD8 | 10:20778226-20779461 | 52.06 | 118.22 | 1.18 | 0.02 |
| NO66 | 10:85324927-85327431 | 1.30 | 3.03 | 1.22 | 0.05 |
| NPC2 | 10:86170652-86179237 | 9.22 | 22.01 | 1.26 | 0.02 |
| NYNRIN | 10:20588781-20600788 | 0.38 | 1.14 | 1.60 | 0.02 |
| PSME1 | 10:20846748-20849410 | 14.09 | 29.91 | 1.09 | 0.02 |
| PSME2 | 10:20838544-20841805 | 16.73 | 35.97 | 1.10 | 0.02 |
| PTGDR | 10:45205472-45212960 | 1.46 | 3.61 | 1.31 | 0.02 |
| RGS2 | 4:58723599-58724929 | 6.20 | 1.93 | -1.68 | 0.02 |
| RPS27L | 10:46946053-46949421 | 76.41 | 149.03 | 0.96 | 0.05 |
| RPS29 | 10:42669270-42671123 | 478.02 | 1185.79 | 1.31 | 0.02 |
| THY1 | 15:30509503-30515399 | 8.94 | 18.91 | 1.08 | 0.02 |

Low-DMI= Low Dry matter intake; High-DMI= High Dry matter intake; FC= log_2_-fold change;

q-value= FDR-adjusted p-value.

**Supplementary Table S5. Gene ontology terms identified as enriched from the DAVID analysis for differentially expressed genes for average daily gain (ADG) between the divergent residual feed intake (RFI) groups**

| Category | Term | PValue | Genes |
| --- | --- | --- | --- |
| GOTERM_BP_FAT | GO:0010033~response to organic substance | 0.01 | FOS, THBS1, MYC, ADIPOQ, PPP1R15A |
| GOTERM_BP_FAT | GO:0007155~cell adhesion | 0.01 | LYVE1, COL22A1, POSTN, THBS1, THBS4, THY1 |
| GOTERM_BP_FAT | GO:0022610~biological adhesion | 0.01 | LYVE1, COL22A1, POSTN, THBS1, THBS4, THY1 |
| GOTERM_BP_FAT | GO:0030198~extracellular matrix organization | 0.02 | CRISPLD2, POSTN, SERPINH1 |
| GOTERM_BP_FAT | GO:0030155~regulation of cell adhesion | 0.02 | COL8A1, THBS1, ADIPOQ |
| GOTERM_BP_FAT | GO:0009611~response to wounding | 0.03 | THBD, PROCR, THBS1, LOC504773 |
| GOTERM_BP_FAT | GO:0043062~extracellular structure organization | 0.03 | CRISPLD2, POSTN, SERPINH1 |
| GOTERM_BP_FAT | GO:0051174~regulation of phosphorus metabolic process | 0.03 | FABP4, RCAN1, THBS1, THY1 |
| GOTERM_BP_FAT | GO:0019220~regulation of phosphate metabolic process | 0.03 | FABP4, RCAN1, THBS1, THY1 |
| GOTERM_BP_FAT | GO:0009968~negative regulation of signal transduction | 0.04 | THBS1, ADIPOQ, THY1 |
| GOTERM_BP_FAT | GO:0010648~negative regulation of cell communication | 0.04 | THBS1, ADIPOQ, THY1 |
| GOTERM_BP_FAT | GO:0009743~response to carbohydrate stimulus | 0.04 | THBS1, ADIPOQ |
| GOTERM_BP_FAT | GO:0009749~response to glucose stimulus | 0.04 | THBS1, ADIPOQ |
| GOTERM_BP_FAT | GO:0034284~response to monosaccharide stimulus | 0.04 | THBS1, ADIPOQ |
| GOTERM_BP_FAT | GO:0009746~response to hexose stimulus | 0.04 | THBS1, ADIPOQ |
| GOTERM_BP_FAT | GO:0007423~sensory organ development | 0.05 | COL8A1, MYC, THY1 |
| GOTERM_BP_FAT | GO:0010605~negative regulation of macromolecule metabolic process | 0.06 | FABP4, THBS1, MYC, ADIPOQ |
| GOTERM_BP_FAT | GO:0032368~regulation of lipid transport | 0.06 | THBS1, ADIPOQ |
| GOTERM_BP_FAT | GO:0050873~brown fat cell differentiation | 0.07 | FABP4, ADIPOQ |
| GOTERM_BP_FAT | GO:0045859~regulation of protein kinase activity | 0.07 | FABP4, THBS1, THY1 |
| GOTERM_BP_FAT | GO:0030203~glycosaminoglycan metabolic process | 0.07 | LYVE1, GLCE |
| GOTERM_BP_FAT | GO:0010811~positive regulation of cell-substrate adhesion | 0.07 | COL8A1, THBS1 |
| GOTERM_BP_FAT | GO:0048584~positive regulation of response to stimulus | 0.07 | FABP4, THBS1, THY1 |
| GOTERM_BP_FAT | GO:0001818~negative regulation of cytokine production | 0.07 | THBS1, ADIPOQ |
| GOTERM_BP_FAT | GO:0043549~regulation of kinase activity | 0.07 | FABP4, THBS1, THY1 |
| GOTERM_BP_FAT | GO:0030336~negative regulation of cell migration | 0.08 | THBS1, THY1 |
| GOTERM_BP_FAT | GO:0051338~regulation of transferase activity | 0.08 | FABP4, THBS1, THY1 |
| GOTERM_BP_FAT | GO:0040013~negative regulation of locomotion | 0.08 | THBS1, THY1 |
| GOTERM_BP_FAT | GO:0048593~camera-type eye morphogenesis | 0.08 | COL8A1, THY1 |
| GOTERM_BP_FAT | GO:0051271~negative regulation of cell motion | 0.08 | THBS1, THY1 |
| GOTERM_BP_FAT | GO:0006022~aminoglycan metabolic process | 0.09 | LYVE1, GLCE |
